# Survival Among Patients With *ERBB2*-Positive Metastatic Breast Cancer and Central Nervous System Disease

**DOI:** 10.1001/jamanetworkopen.2024.57483

**Published:** 2025-01-31

**Authors:** Emanuela Ferraro, Anne S. Reiner, Rabih Bou Nassif, Umberto Tosi, Samantha Brown, Sabrina Zeller, Chau T. Dang, Andrew D. Seidman, Nelson S. Moss

**Affiliations:** 1 Breast Medicine Service, Department of Medicine, Memorial Sloan Kettering Cancer Center, New York, New York; 2 Department of Epidemiology and Biostatistics, Memorial Sloan Kettering Cancer Center, New York, New York; 3 Department of Neurosurgery and Brain Metastasis Center, Memorial Sloan Kettering Cancer Center, New York, New York

## Abstract

**Importance:**

Approximately one-third of patients with *ERBB2* (formerly *HER2* or *HER2/neu*)–positive (*ERBB2*^+^) metastatic breast cancer (MBC) develop brain metastasis. It is unclear whether patients with disease limited to the central nervous system (CNS) have different outcomes and causes of death compared with those with concomitant extracranial metastasis.

**Objective:**

To assess overall survival (OS) and CNS-related mortality among patients with *ERBB2*^+^ breast cancer and a diagnosis of CNS disease by disease distribution (CNS only vs CNS plus extracranial metastasis).

**Design, Setting, and Participants:**

This single-center, retrospective cohort study included patients with *ERBB2*^+^ MBC and CNS disease, including parenchymal brain metastasis, leptomeningeal disease (LMD), or dural metastasis, who were treated between August 2010 and April 2022 at Memorial Sloan Kettering Cancer Center. Data were analyzed between December 2023 and August 2024.

**Main Outcomes and Measures:**

Overall survival, estimated with the Kaplan-Meier method, and CNS-related mortality with cumulative incidence.

**Results:**

The cohort included 274 patients (272 [99.3%] female). The median age was 53.7 years (range, 28.7-87.4 years); 125 patients (45.6%) presented with de novo MBC. At CNS metastasis diagnosis, 73 (26.6%) presented with CNS-only disease. There was a median follow-up of 3.7 years (range, 0.2-12.0 years) from CNS disease diagnosis among those alive at the end of follow-up. Both OS and CNS-related death were significantly correlated with the pattern of presentation: OS was shortest among patients with LMD (1.24 years; 95% CI, 0.89-2.08 years) followed by those with extracranial metastasis (2.16 years; 95% CI, 1.87-2.58 years) and was longest among patients with parenchymal or dural CNS disease only (3.57 years; 95% CI, 2.10-5.63 years) (*P* = .001). Of 192 patients (70.1%) who died, 106 (55.2%) died of a CNS-related cause. The group with CNS-only disease remained at high risk of death from CNS causes, with a 3-year CNS-related death rate of 33.98% (95% CI, 22.84%-45.43%) and a 3-year death rate from other causes of 6.07% (95% CI, 1.93%-13.69%). On multivariable modeling for CNS-related death, LMD (hazard ratio, 1.87; 95% CI, 1.19-2.93; *P* = .007) and treatment with whole-brain radiotherapy (hazard ratio, 1.71; 95% CI, 1.13-2.58; *P* = .01) were associated with CNS-related death.

**Conclusions and Relevance:**

In this cohort study, 55.2% of deaths among patients with *ERBB2*^+^ breast cancer and brain metastasis were due to CNS-related causes, with the greatest risk among patients with LMD. CNS-only presentation was associated with improved survival but a higher rate of CNS-related death, supporting an approach of aggressive local therapy for select patients.

## Key Points

**Question:**

What are the overall and central nervous system (CNS)–related mortality rates among patients with *ERBB2*-positive metastatic breast cancer with CNS disease only compared with those with both CNS and extracranial metastasis?

**Findings:**

In this cohort study of 274 patients with metastatic *ERBB2*-positive breast cancer and CNS metastasis, those with CNS-only disease had longer overall survival and a lower risk of death from any cause compared with patients with concomitant extracranial metastases.

**Meaning:**

The findings suggest that aggressive treatments (both local and systemic) may be warranted to control the intracranial progression of CNS metastases.

## Teaser

This cohort study explores survival among patients with *ERBB2*-positive metastatic breast cancer with only central nervous system (CNS) metastasis compared with CNS and extracranial metastases.

## Introduction

Brain metastasis is a common and feared complication of breast cancer, affecting approximately 30% to 35% of patients with advanced *ERBB2* (formerly *HER2* or *HER2/neu*)–positive (*ERBB2*^+^) disease.^1^ In the past decade, improving therapies have led to higher rates of durable response and significant survival benefit in *ERBB2*^+^ metastatic breast cancer (MBC). For example, first-line pertuzumab and trastuzumab plus chemotherapy improved median survival to over 5 years, and similar results have been obtained for second-line treatment with the antibody-drug conjugate trastuzumab deruxtecan (T-DXd).^1,2,3^ However, the efficacy of many of these agents is hypothesized to be driven by anticancer activity in the extracranial compartment rather than the central nervous system (CNS), particularly for antibody-based therapy, owing to the blood-brain tumor barrier, which partially excludes macromolecules from the CNS compartment. For example, the addition of pertuzumab to trastuzumab and docetaxel delayed but did not reduce the risk of CNS progression in the CLEOPATRA study.^4^ As a result, the incidence and impact of brain metastasis in patients with *ERBB2*^+^ MBC are expected to increase.

Therapies effective against *ERBB2*^+^ brain metastasis are thus an urgent and unmet need, and there has been an increasing focus on reporting CNS outcomes in clinical trials. Recently, CNS response and intracranial progression-free survival benefit were observed with both T-DXd and tucatinib as second- and third-line treatment.^5,6^ While extracranial disease can in some cases be durably controlled with these regimens, CNS metastases remain challenging to treat, necessitating CNS-directed local therapies, including radiotherapy and/or neurosurgical resection, in most cases.^7^ These multimodal local approaches offer a high rate of local disease control and symptomatic palliation for appropriate patients, but they also carry potential risks (eg, systemic treatment breaks) and have unclear effects on overall survival (OS) and CNS-related mortality.^8,9,10,11^

While CNS metastases are uniquely disabling, there are limited data on the cause of death for such patients, in part owing to the complexity and heterogeneity of advanced disease.^12^ However, understanding the rate of CNS-related death and its potential correlates may help determine which patients might most benefit from aggressive local therapies, including radiation and/or craniotomy. We sought to identify the contemporary risk of CNS-related death among patients with brain metastasis, hypothesizing that disease extent between intracranial and extracranial compartments would directly inform potential risk of death and affect OS.

## Methods

This single-center, retrospective cohort study was performed at Memorial Sloan Kettering Cancer Center, which gave institutional review board approval and waived patient informed consent given the retrospective nature of the study. This study was conducted in accordance with the Good Clinical Practice guidelines and the Declaration of Helsinki.^13^ Medical records of consecutive patients diagnosed with *ERBB2*^+^ MBC and CNS metastasis (including parenchymal brain metastasis, leptomeningeal disease [LMD], and/or dural metastasis) at any time during their clinical course between August 2010 and April 2022 were included. The analyses were performed between December 2023 and August 2024. This study followed the Strengthening the Reporting of Observational Studies in Epidemiology (STROBE) reporting guideline. *ERBB2* positivity was defined by protein overexpression or by gene amplification according to the 2018 American Society of Clinical Oncology (ASCO) and College of American Pathologists guidelines, as performed on metastatic tissue samples when available.^14^

Clinical and radiologic data were collected. Self-reported race and ethnicity were retrieved from electronic health records and were included in the analysis as we were exploring whether these are potential risk factors for CNS-related death. Categories were Asian, Black, White, other (American Indian or Alaska Native, Pacific Islander), and unknown. The time of CNS disease diagnosis was defined as the date of the first cross-sectional brain imaging (magnetic resonance imaging [MRI] or computed tomography [CT]) showing brain metastasis. Brain imaging was typically performed for a symptomatic concern or to characterize suspicious findings partially captured on extracranial imaging. The LMD diagnosis date was defined by the first positive cerebrospinal fluid cytology result or circulating tumor cell detection and/or contrast-enhanced brain or spinal MRI showing dissemination. CNS imaging was reviewed by dedicated neuroradiologists. Extracranial disease status at CNS metastasis diagnosis and last follow-up was collected from available imaging reports (chest, abdomen, and/or pelvis CT, fludeoxyglucose-18 positron emission tomography, and/or bone scan). Neurologic symptoms at diagnosis and last follow-up or death were reviewed by 2 independent investigators (R.B.N., U.T.) with expertise in neurologic examination.

The study population was categorized into 2 groups: (1) patients with CNS metastasis only, defined as no evidence of extracranial metastasis (either never previously developed or prior extracranial metastasis with subsequent complete response), and (2) patients with concomitant extracranial metastasis (stable or progressing disease). We used 31 days as a temporal cutoff to define synchronous disease, extracranial metastasis first, and CNS metastasis first.

The primary objective was to assess the proportion of patients with *ERBB2*^+^ MBC and brain metastasis who died of CNS-related or other causes. CNS-related death was defined as any death from brain metastasis and/or leptomeningeal progression; these deaths were further subcategorized as death from tumor-related brain injury (eg, elevated intracranial pressure and herniation), functional deficits and/or seizures, or complications of brain-directed treatment (eg, radiation necrosis). Neurologic impairment secondary to other causes (ie, hepatic or uremic encephalopathy) was considered non–CNS related. Death from other causes included extracranial disease progression and death from nononcologic causes.

### Statistical Analysis

The cohort was characterized using descriptive statistics, including proportions, medians, and ranges. Time from breast cancer diagnosis to CNS metastasis by disease type was compared using the Kruskal-Wallis test. Overall survival and rate of CNS-related death were calculated from the date of CNS disease diagnosis until death or last follow-up for censored patients, with OS assessed using the Kaplan-Meier method. Cox proportional hazards regression modeling identified variables associated with OS. CNS-related death rates and treatment rates were estimated using cumulative incidence as a competing risk (with death due to other causes as a competing event). Potential factors associated with CNS-related death were analyzed using subdistribution hazards regression, including baseline variables (age; metastasis type and number at CNS diagnosis; *ERBB2*, estrogen receptor, and progesterone receptor status; and disease pattern at the diagnosis of MBC) and time-dependent treatment variables (medical treatments, whole-brain radiotherapy [WBRT], and/or stereotactic radiosurgery [SRS]). Tests were 2-sided with statistical significance set at *P* <  .05. Analyses were performed in SAS, version 9.4 (SAS Institute, Inc) and R, version 4.3.2 (R Project for Statistical Computing).

## Results

### Clinicopathologic Characteristics of the Study Population

A total of 293 patients with *ERBB2*^+^ MBC and brain metastasis were identified (eFigure 1 in Supplement 1). Five patients were excluded due to a prior or concomitant second malignant neoplasm without pathologic confirmation of brain metastasis, 11 due to discordant primary metastatic *ERBB2* status or 1 *ERBB2*-negative metastatic site and no receipt of anti-*ERBB2* therapy for metastasis, and 3 due to having no clinical data regarding local and systemic therapies and no details of their disease distribution. Thus, 274 patients were included in the analysis. Patients’ characteristics are summarized in Table 1. Almost all patients were female (272 [99.3%]); 2 (0.7%) were male. The median age at CNS metastasis diagnosis was 53.7 years (range, 28.7-87.4 years). Twenty-seven patients (9.9%) were Asian, 29 (10.6%) were Black, 185 (67.5%) were White, 12 (4.4%) reported other race and ethnicity, and 21 (7.7%) had unknown race and ethnicity. Overall, 125 patients (45.6%) presented with de novo MBC, while the remainder (149 [54.4%]) had a prior diagnosis of early-stage breast cancer. The median time from initial breast cancer diagnosis to CNS metastasis identification was 32.2 months (range, 0-183.0 months), with a median of 1 line (range, 0-14 lines) of systemic therapy before brain metastasis. For patients with extracranial metastasis as the first site of metastatic disease (156 [56.9%]), CNS metastasis developed after a median of 41.6 months (range, 3.3-183.0 months) from breast cancer diagnosis; patients who developed CNS disease as the first metastatic recurrence (64 [23.4%]) and patients with synchronous recurrence (52 [19.0%]) had similar time to CNS disease (25.0 months [range, 0.9-81.2 months] and 24.6 months [range, 0-105.8 months], respectively; *P* < .001) (eFigure 2 in Supplement 1).

**Table 1.** Patient and Disease Characteristics

Characteristic	Patients (N = 274)^a^
Age at CNS metastasis, median (range), y	53.7 (28.7-87.4)
Sex	
Female	272 (99.3)
Male	2 (0.7)
Race and ethnicity	
Asian	27 (9.9)
Black	29 (10.6)
White	185 (67.5)
Other^b^	12 (4.4)
Unknown	21 (7.7)
Stage at initial diagnosis	
Early stage (I-III)	149 (54.4)
De novo metastatic (IV)	125 (45.6)
ER and/or PR status	
Positive	147 (53.6)
Negative	127 (46.4)
*ERBB2*^+^ status site	
Primary only	10 (3.6)
Metastasis	264 (96.4)
*ERBB2* status details	
IHC 3^+^	214 (78.1)
IHC 2^+^, FISH amplified	33 (12.0)
Unknown	27 (9.9)
Disease distribution at CNS disease diagnosis	
CNS only	73 (26.6)
Extracranial metastasis and CNS	199 (72.6)
Unknown	2 (0.7)
Type of metastases at CNS disease diagnosis	
LMD at brain metastasis diagnosis	48 (17.5)
Only brain parenchymal or dural at brain metastasis diagnosis	54 (19.7)
Extracranial metastasis before or concurrent with brain metastasis diagnosis	170 (62.0)
Unknown	2 (0.7)
Extracranial metastasis status at CNS disease diagnosis	
Complete response	9 (3.3)
No POD	67 (24.5)
POD	133 (48.5)
Never developed extracranial metastasis	57 (20.8)
Unknown	8 (2.9)
Parenchymal brain metastasis at diagnosis, No.	
0	15 (5.5)
1	71 (25.9)
2-3	64 (23.4)
>3	124 (45.3)
Dural metastases at diagnosis, No.	
1	26 (9.5)
≥2	10 (3.6)
LMD at CNS diagnosis	
Same date as CNS diagnosis	43 (15.7)
Within 1 mo of CNS diagnosis	5 (1.8)
Symptoms at CNS disease diagnosis	
Altered mental status	7 (2.6)
Dizziness or vague symptoms	68 (24.8)
Focal	47 (17.2)
ICP or mass effect with or without other symptoms	58 (21.2)
Seizure	28 (10.2)
No symptoms	58 (21.2)
Unknown	8 (2.9)
Anti-*ERBB2* therapy in early stage	
Adjuvant	64 (23.4)
Neoadjuvant	92 (33.6)
None	118 (43.1)
Prior *ERBB2* antibody and taxane therapy	
Yes	241 (88.0)
No	32 (11.7)
Unknown	1 (0.4)
Treatment lines before CNS disease diagnosis	
Median (range), No.	1 (0-14)
Unknown	2 (0.7)
Treatment lines after CNS disease diagnosis	
Median (range), No.	1 (0-15)
Unknown	12 (4.4)
First TKI after CNS disease diagnosis	
Lapatinib	63 (23.0)
Neratinib	10 (3.6)
Tucatinib	38 (13.9)
None	149 (54.4)
Unknown	14 (5.1)
First ADC after CNS diagnosis	
T-DM1	67 (24.4)
T-DXd	19 (6.9)
Unknown	14 (5.1)
Local treatment	
SRS	110 (40.1)
WBRT	102 (37.2)
Both SRS and WBRT	42 (15.3)
Surgery	78 (28.5)

Abbreviations: ADC, antibody drug conjugate; CNS, central nervous system; ER, estrogen receptor; FISH, fluorescent in situ hybridization; ICP, intracranial pressure; IHC, immunohistochemistry; LMD, leptomeningeal disease; POD, progression of disease; PR, progesterone receptor; SRS, stereotactic radiosurgery; T-DM1, trastuzumab emtansine; T-DXd, trastuzumab deruxtecan; TKI, tyrosine kinase inhibitor; WBRT, whole-brain radiation therapy.

^a^ Data are presented as number (percentage) of patients unless otherwise indicated.

^b^ Includes American Indian or Alaska Native and Pacific Islander.

### CNS Disease Burden and Treatments

At CNS metastasis diagnosis, 73 patients (26.6%) presented with CNS-only disease (64 [87.7%] without a prior extracranial metastasis diagnosis and 9 [12.3%] with a prior extracranial metastasis diagnosis who had experienced complete response) and 199 (72.6%) with concomitant extracranial metastasis. Two patients (0.7%) had unknown status of extracranial disease at diagnosis of CNS disease. Among the 73 patients with CNS-only disease, 33 (45.2%) never subsequently developed extracranial metastasis progression by last follow-up or death. Most patients had parenchymal brain metastasis (259 [94.5%]), of whom 41 (15.8%) had concomitant LMD at CNS diagnosis. Ten patients (3.6%) presented with dural metastasis only and 5 (1.8%) with LMD only. Among patients with parenchymal brain metastasis, 71 of 259 (27.4%) presented with a solitary brain lesion, 64 (24.7%) had 2 or 3 lesions, and the remainder (123 [47.5%]) had more than 3 lesions.

Nearly all patients (254 [92.7%]) were treated with CNS-directed radiotherapy: 110 of 274 (40.1%) received SRS only, 102 of 274 (37.2%) received WBRT only, and 42 of 274 (15.3%) received both. Of the 20 patients not receiving WBRT or SRS, 1 (5.0%) received proton therapy, 1 (5.0%) declined treatment, and 1 (5.0%) had small, asymptomatic lesions for which treatment was deferred by the physician. Three of these 20 patients (15.0%) were ineligible for local treatment due to rapid performance status decline, 2 (10.0%) had LMD, and 1 (5.0%) had dural metastasis for which local therapy was not recommended. Data on the reasons for radiation treatment decisions for the remaining 11 of 20 patients (55.0%) were unavailable. Overall, 89 of 274 patients (32.5%) underwent at least 1 neurosurgical resection, and 14 (5.1%) underwent more than 1. Almost all patients (242 [88.3%]) were exposed to taxanes and anti-*ERBB2* antibodies prior to CNS disease development. Following CNS metastasis diagnosis, 111 patients (40.5%) received at least 1 *ERBB2* tyrosine kinase inhibitor (TKI), and 86 (31.4%) received at least 1 *ERBB2*-directed antibody drug conjugate (ADC). Among the agents with demonstrated higher CNS efficacy, tucatinib was given to 48 patients (17.5%) and T-DXd to 32 (11.7%).

Patients with CNS metastasis only were more likely over time to be treated with brain metastasis surgery compared with patients with both CNS and extracranial metastases (3-year rate, 49.46% [95% CI, 37.42%-60.40%] vs 19.37% [95% CI, 14.15%-25.21%]; *P* < .001); baseline characteristics for the 2 groups are presented in the eTable in Supplement 1. No other statistically significant differences in terms of local treatment interventions were observed between the 2 groups.

### CNS-Related Death and Clinical Factors

**Figure 1.**
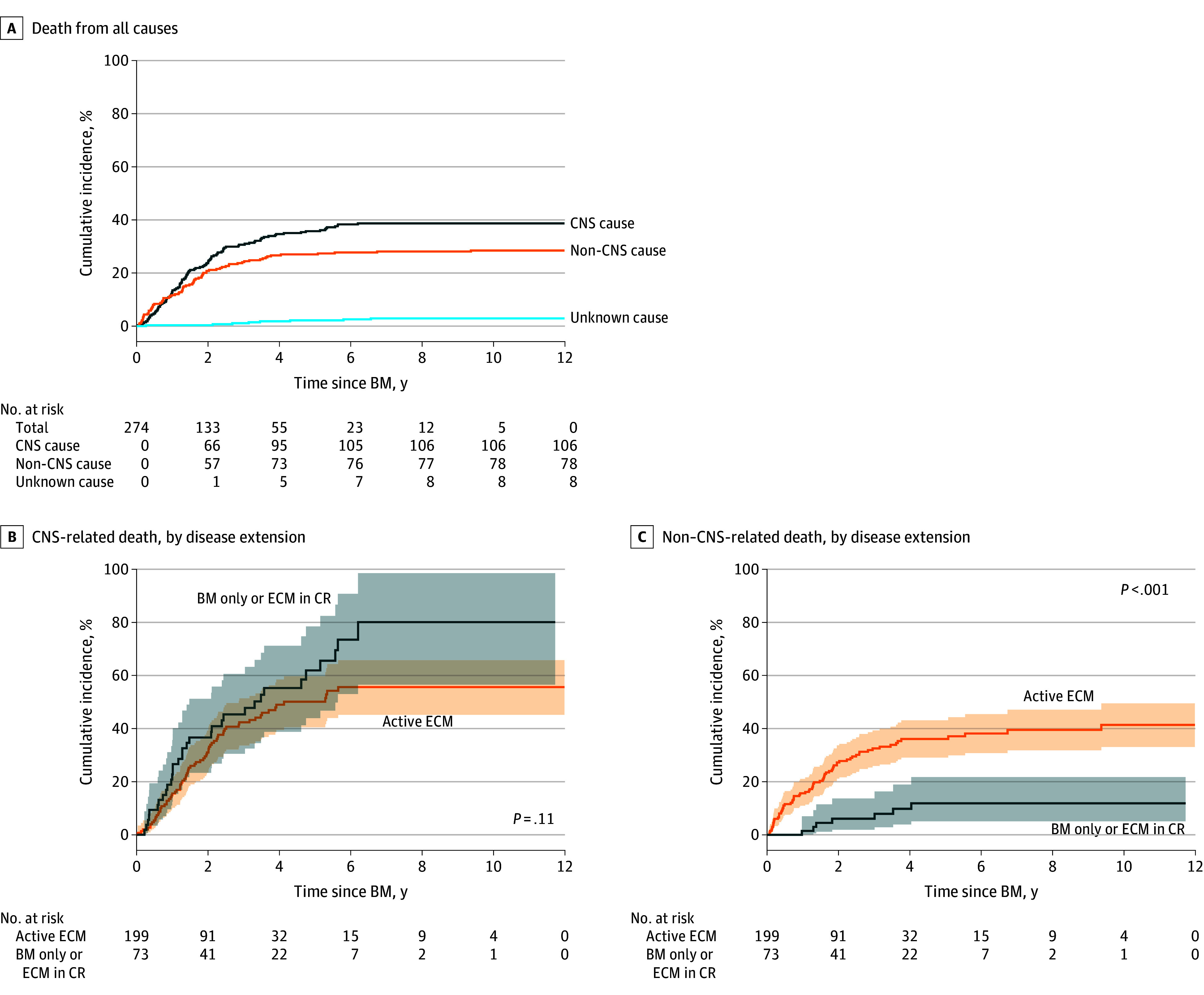
Cumulative Incidence of Death by Time Since Diagnosis of Central Nervous System (CNS) Metastasis BM indicates brain metastasis; CR, complete response; ECM, extracranial metastasis.

Overall, 192 patients died (70.1%); 106 of these deaths (55.2%) were due to a CNS-related cause. The most common cause of CNS-related death was brain metastasis progression–associated decline, which occurred in 72 of the 192 cases (37.5%) (Table 2). The decline occurring with untreatable brain metastasis progression included several complications as consequences of the CNS involvement, such as focal deficits, acute intracranial hypertension or mass effect, refractory seizure, or progressive loss of functional status and vital functions. Development of LMD and treatment with trastuzumab emtansine (T-DM1), TKI, or WBRT following CNS disease diagnosis were associated with CNS-related death in both univariable and multivariable models (eg, multivariable model for TKI: hazard ratio [HR], 1.94 [95% CI, 1.27-2.97]; *P* = .002; T-DM1: HR, 1.95 [95% CI, 1.21-3.13]; *P* = .006; WBRT: HR, 1.71 [95% CI, 1.13-2.58]; *P* = .01) (Table 3). The cumulative incidence of CNS-related death at 3 years was 32.52% (95% CI, 26.84%-38.32%) (Figure 1A) and did not differ significantly between patients with CNS metastasis only and those with CNS metastasis and extracranial metastasis (33.98% [95% CI, 22.84%-45.43%] vs 31.76% [95% CI, 25.19%-38.52%]; *P* = .11) (Figure 1B). Death due to other causes was significantly more likely to occur in the group of patients with CNS metastasis and extracranial metastasis (3-year rate, 32.55%; 95% CI, 25.94%-39.31%) than in the group with CNS metastasis only (3-year rate, 6.07%; 95% CI, 1.93%-13.69%) (*P* < .001) (Figure 1C).

**Table 2.** Causes of Death

Cause	Patients, No. (%) (n = 192)
CNS cause of death	
Any	106 (55.2)
Brain metastasis progression	
Overall	72 (37.5)
Increase in No. of lesions	32 (16.6)
Increase in size of known lesions (tumor or edema)	26 (13.5)
Local progression in the surgery bed	5 (2.6)
Clinical progression^a^	7 (3.6)
Increase in size due to radionecrosis	2 (1.0)
Intratumoral hemorrhage	3 (1.6)
LMD progression (with or without parenchymal progression)	30 (15.6)
Stroke	1 (0.5)
Non-CNS cause of death	86 (44.8)

Abbreviations: CNS, central nervous system; LMD, leptomeningeal disease.

^a^ Includes intracranial hypertension, ataxia, seizure, and neurologic decline.

**Table 3.** Univariable and Multivariable Associations With CNS-Related Death

Variable	Univariable model		Multivariable model	
	HR (95% CI)	*P* value	HR (95% CI)	*P* value
LMD^a^	2.32 (1.53-3.53)	<.001	1.87 (1.19-2.93)	.007
WBRT^a^	2.00 (1.35-2.96)	<.001	1.71 (1.13-2.58)	.01
SRS alone^a^	0.71 (0.47-1.06)	.09	NA	NA
Surgery^a^	0.88 (0.58-1.35)	.56	NA	NA
T-DM1^a^	2.08 (1.37-3.16)	<.001	1.95 (1.21-3.13)	.006
T-DXd	1.39 (0.58-3.37)	.46	NA	NA
TKI^a^	2.58 (1.76-3.80)	<.001	1.94 (1.27-2.97)	.002
Tucatinib	0.86 (0.42-1.78)	.68	NA	NA
Any RT^a^	3.41 (1.38-8.41)	.008	NA	NA
Treatment lines before brain metastasis diagnosis	0.94 (0.86-1.03)	.18	NA	NA
Age at brain metastasis	1.01 (1.00-1.03)	.13	NA	NA
Type of metastasis at brain metastasis diagnosis				
Extracranial metastasis	1 [Reference]	NA	1 [Reference]	NA
Only brain metastasis	1.52 (1.00-2.29)	.048	1.42 (0.92-2.20)	.12
Parenchymal metastases, No.				
0	1 [Reference]	NA	NA	NA
1	0.53 (0.22-1.27)	.15	NA	NA
2	0.58 (0.23-1.47)	.25	NA	NA
≥3	0.80 (0.35-1.83)	.60	NA	NA
Dural metastases, No.				
0	1 [Reference]	NA	NA	NA
1	0.85 (0.42-1.72)	.65	NA	NA
2	0.41 (0.08-2.24)	.31	NA	NA
≥3	0.41 (0.07-2.48)	.33	NA	NA
De novo metastases				
No	1 [Reference]	NA	NA	NA
Yes	0.96 (0.66-1.39)	.81	NA	NA
*ERBB2* antibodies and taxanes before brain metastasis				
No	1 [Reference]	NA	NA	NA
Yes	0.86 (0.50-1.45)	.56	NA	NA
ER and PR status of metastasis^b^				
Negative	1 [Reference]	NA	NA	NA
Positive	0.97 (0.66-1.41)	.86	NA	NA
*ERBB2* status of metastasis^b^				
Negative	1 [Reference]	NA	NA	NA
Positive	1.19 (0.35-4.04)	.78	NA	NA
Extracranial metastasis disease status at time of brain metastasis				
Complete response	1 [Reference]	NA	NA	NA
No diagnosis of ECD	2.16 (0.59-7.93)	.25	NA	NA
No POD	1.84 (0.50-6.71)	.36	NA	NA
POD	1.31 (0.36-4.75)	.68	NA	NA

Abbreviations: CNS, central nervous system; ECD, extracranial disease; ER, estrogen receptor; HR, hazard ratio; LMD, leptomeningeal disease; NA, not applicable; POD, progression of disease; PR, progesterone receptor; RT, radiotherapy; SRS, stereotactic radiosurgery; T-DM1, trastuzumab emtansine; T-DXd, trastuzumab deruxtecan; TKI, tyrosine kinase inhibitor; WBRT, whole-brain radiation therapy.

^a^ Time varying.

^b^ First site of extracranial metastasis or brain metastasis.

### Survival Analysis

**Figure 2.**
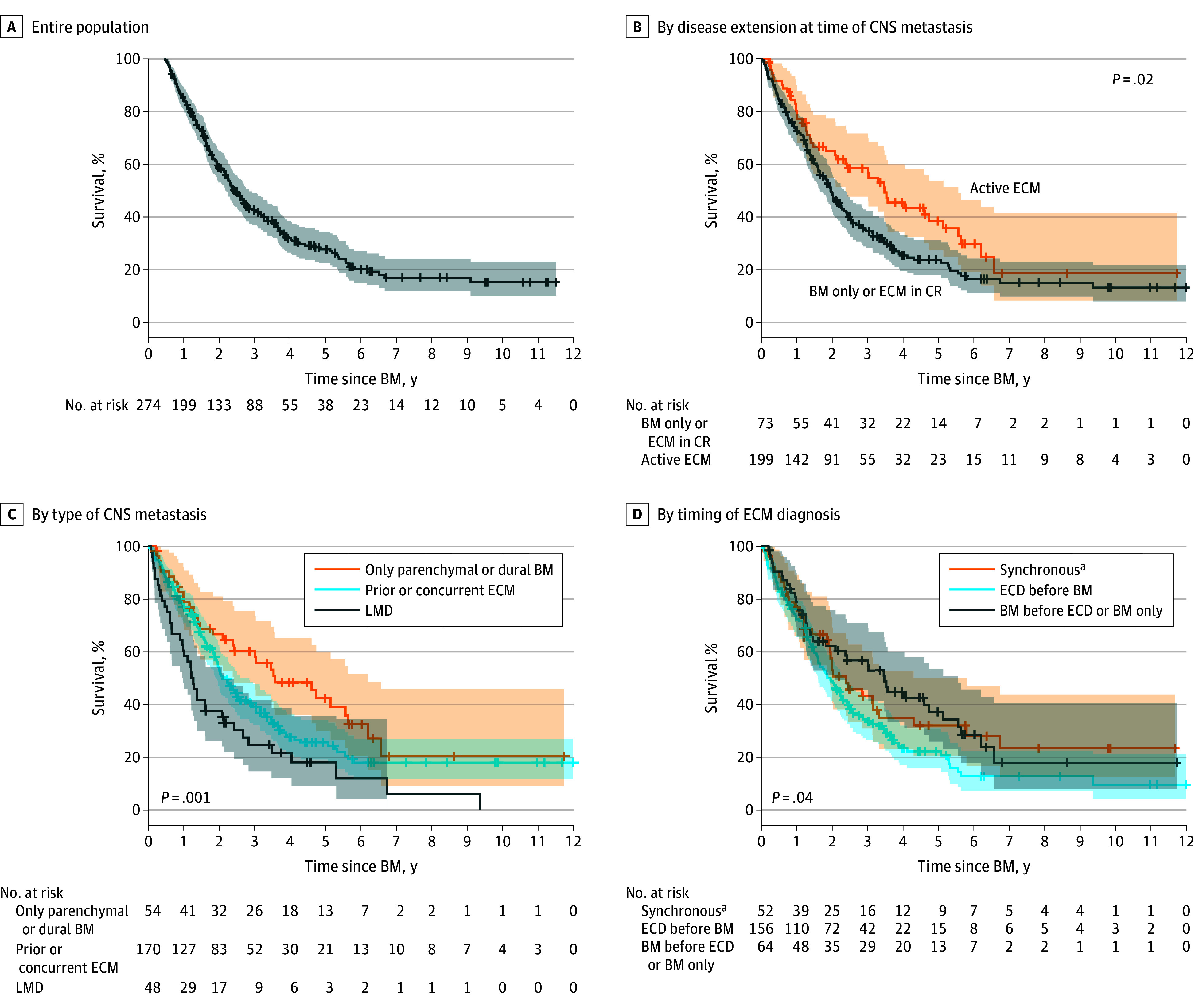
Overall Survival After Diagnosis of Central Nervous System (CNS) Metastasis Hash marks represent censoring. BM indicates brain metastasis; CR, complete response; ECD, extracranial disease; ECM, extracranial metastasis; LMD, leptomeningeal disease. ^a^Within 1 month.

After a median follow-up of 3.7 years (range, 0.2-12.0 years) among those alive at the end of follow-up, the median OS in the full cohort was 2.13 years (95% CI, 1.86-2.50 years) (Figure 2A). Patients who presented with CNS-only disease had significantly longer OS of 3.48 years (95% CI, 2.10-5.14 years) compared with 1.96 years (95% CI, 1.62-2.27 years) for patients with concomitant extracranial metastasis (*P* = .02), with a 5-year OS of 38.43% (95% CI, 25.50%-51.36%) vs 23.70% (95% CI, 17.09%-30.31%), attributable to extracranial causes of death (Figure 2B). When the model was adjusted for timing of disease presentation (synchronous, extracranial metastasis first, and CNS first) and type of CNS metastasis, patients with both CNS metastasis and extracranial metastasis did not have worse OS than patients with CNS-only disease (HR, 2.66; 95% CI, 0.84-8.36; *P* = .10), while LMD diagnosis was independently associated with CNS-related death (HR, 1.87; 95% CI, 1.19-2.93; *P* = .007) and shorter OS (HR, 3.13; 95% CI, 1.15-8.48; *P* = .03). Univariate analysis of CNS metastasis type identified prolonged OS among patients with parenchymal brain metastasis or dural metastasis only (3.57 years; 95% CI, 2.10-5.63 years) compared with patients with parenchymal brain metastasis and extracranial metastasis (2.16 years; 95% CI, 1.87-2.58 years) and patients with LMD with or without other metastases (1.24 years; 95% CI, 0.89-2.08 years) (*P* = .001) (Figure 2C). Overall survival by timing of presentation of extracranial metastasis and CNS metastases also differed between patients with synchronous (2.39 years; 95% CI, 1.86-3.32 years), metachronous extracranial metastasis–first (1.96 years; 95% CI, 1.60-2.27 years), and CNS-first (3.48 years; 95% CI, 1.83-5.14 years) disease (*P* = .04), with patients with metachronous extracranial metastasis–first disease experiencing the worst OS (Figure 2D).

## Discussion

Despite marked improvements in survival among patients with *ERBB2*^+^ breast cancer, CNS recurrence remains a common issue, as most drugs lack effective CNS penetration.^15^ Thus, an emerging problem is the increasing number of patients with CNS-limited disease, as highlighted by our study, which identified 26.6% of patients presenting with CNS-only disease at CNS metastasis diagnosis. Of those, 45.2% never experienced extracranial progression and had a reduced overall mortality rate compared with those with preceding, concomitant, or subsequent extracranial progression. These findings underscore that patients with CNS-only disease are a distinct group of patients characterized by a unique clinical course. Since most patients in this group had CNS metastasis as their first site of distant disease recurrence, we believe that systemic treatments contributed to prevent extracranial progression, leading to prolonged OS. This highlights the need to develop new strategies to treat and/or prevent intracranial progression, including new drugs able to penetrate the CNS and combined with local approaches.

Our results are in contrast with a recent cohort study by Noteware et al^16^ in which patients with CNS-only disease had worse OS than patients with concomitant extracranial metastasis. These discordant results may be due to differences in patient characteristics. First, in our study, 88.3% of patients received taxane and anti-*ERBB2* therapy prior to development of CNS metastases compared with only half of patients in the study by Noteware et al,^16^ potentially impacting the control of micrometastatic disease and survival. In contrast to the study by Noteware et al,^16^ which focused on patients treated with radiation, a substantial feature of our study was the extensive use of neurosurgery as a key component of the treatment strategy for patients with limited (≤3) metastases. In patients with solitary brain metastasis of any solid tumor, surgery followed by radiation has been reported as an effective treatment strategy for local control, with still unclear benefit on survival.^8,17^ As previously shown by our group in a cohort of patients with solid tumors and oligometastatic CNS disease treated with surgery and SRS in the clinical setting, the median OS was 18 months, significantly longer than that among historical controls.^18^ This paradigm supports the ASCO recommendation for maintaining systemic treatment in the context of CNS-only progression.^7^

In our study, the estimated 3-year rate of CNS-related death was 32.52%, which is similar to what was previously reported by Niikura et al,^19^ who identified a CNS-related death rate of approximately 40% among Asian patients with MBC and *ERBB2*^+^ brain metastasis likely treated with trastuzumab only or no anti-*ERBB2* agents. Although most of the patients in our study were pretreated with anti-*ERBB2* agents, the CNS-related death rate was higher than that reported for other cancers.^20^ In a large, single-center study including more than 400 patients with solid tumors and brain metastasis in the clinical setting, only 10% of patients died of CNS-related causes.^20^ We believe that the significant discordance in *ERBB2*^+^ MBC is related to how this disease responds to systemic therapies in the extracranial compartment, resulting in a higher proportion of patients with disease confined to the brain. For example, patients receiving first-line trastuzumab and pertuzumab–based therapy achieved radiologically complete response in one-fourth of cases.^21^ This rate was even higher among patients with de novo metastatic *ERBB2*^+^ disease in the cohort in our study (45.6% of the population and more than double the expected proportion among patients with *ERBB2*^+^ breast cancer^22^). In addition, the aforementioned pancancer study^20^ included a sizable proportion with lung cancer, which is associated with a substantial extracranial disease burden. Additionally, the aforementioned study^20^ included patients referred for SRS who may have had a more limited CNS disease burden compared with the cohort in the current study. The cohort in our study included all patients receiving care at our center regardless of the treatment received, reflecting a population with a higher disease burden.

In terms of factors associated with CNS-related death, WBRT was an independent factor while SRS was not, which may reflect refractoriness to other systemic or less toxic local therapies, more rapid disease progression, or treatment-related toxicity. LMD was independently associated with CNS-related death and shorter OS, as has been previously demonstrated.^23,24,25^ Limited data on new anti-*ERBB2* agents among patients with LMD are available, and our findings support the urgent clinical need for clinical trials focusing on this population in the prevention, screening, and treatment domains.

We believe that the association we found between post–brain metastasis TKI exposure (excluding tucatinib) and CNS-related death reflects attempts by treating physicians to bypass the blood-brain barrier with these small molecules when the predominant disease burden is in the CNS rather than extracranial. In this study, TKIs more frequently received were neratinib and/or lapatinib, which exhibit relatively low CNS activity. When tucatinib was analyzed separately, an association with CNS-related death was not found, aligning with the demonstrated OS benefit of tucatinib in the HER2-CLIMB study.^26^ With respect to ADCs, patients who received T-DM1 appeared to have a higher risk of CNS-related death, in contrast with a clinical trial showing signs of intracranial activity of T-DM1.^27^ This finding may be attributed to the early diagnosis of CNS disease in the present cohort, with a median of only 1 line of systemic therapy before metastasis. Conversely, the limited cohort of T-DXd–treated patients did not appear have a higher risk of CNS-related death, consistent with the pooled analysis of the DESTINY-Breast 01, 02, and 03 studies, in which CNS efficacy of this agent was observed in the subgroup of patients with stable or active brain metastasis.^28^ With emerging effective CNS-penetrant systemic therapies, studies are needed to optimize multimodal CNS-directed treatments to maximize efficacy while minimizing toxicity. For example, late radiotherapy toxicity is increasingly relevant for patients who are living longer, and concomitant SRS and ADC therapy may pose significant additional risk over temporally spaced treatments.^10,29^

### Limitations

Our study had some limitations. This was a retrospective study conducted at a single cancer center with a dedicated multidisciplinary brain metastasis program, clinical trial options, and a high volume of surgical and radiotherapy referrals.^30,31^ In addition, the definition of CNS-related death was based on dominant symptom patterns at near end-of-life evaluations. However, despite the intrinsic difficulty of ascertaining cause of death retrospectively, we believe our definition is a good surrogate for death related to brain metastasis and its sequelae and was similar to that used in other published analyses.^20^ Due to the intrinsically retrospective nature of the study, neurologic outcomes and quality-of-life parameters were not evaluated.

## Conclusions

In this cohort study of 274 patients with *ERBB2*^+^ MBC and brain metastasis, CNS progression was the most common cause of death. Despite high rates of local therapy, patients with CNS-only disease experienced longer survival than those with concomitant extracranial metastasis but still had significant CNS-related mortality. More effective CNS-penetrant systemic therapies are urgently needed. As new anti-*ERBB2* and other anticancer agents emerge, clinical trials should include patients with CNS disease to evaluate intracranial efficacy from the early stages of drug development. Additionally, trial designs should incorporate end points that specifically address CNS outcomes, including CNS-related mortality.

## Notes

Supplement 1. **eTable.** Clinicopathologic Characteristics Associated With the Presence of ECM Prior to or Synchronous With CNS Disease at Metastatic Disease Diagnosis **eFigure 1.** Flow Diagram **eFigure 2.** Time to Development of CNS Disease From the Diagnosis of Primary Breast Cancer

Supplement 2.
Data Sharing Statement

## References

[1] Kuksis M, Gao Y, Tran W, . The incidence of brain metastases among patients with metastatic breast cancer: a systematic review and meta-analysis. Neuro Oncol. 2021;23(6):894-904. doi:10.1093/neuonc/noaa285 33367836 PMC8168821

[2] Cortés J, Kim SB, Chung WP, ; DESTINY-Breast03 Trial Investigators. Trastuzumab deruxtecan versus trastuzumab emtansine for breast cancer. N Engl J Med. 2022;386(12):1143-1154. doi:10.1056/NEJMoa2115022 35320644

[3] Baselga J, Cortés J, Kim SB, ; CLEOPATRA Study Group. Pertuzumab plus trastuzumab plus docetaxel for metastatic breast cancer. N Engl J Med. 2012;366(2):109-119. doi:10.1056/NEJMoa1113216 22149875 PMC5705202

[4] Swain SM, Baselga J, Miles D, . Incidence of central nervous system metastases in patients with HER2-positive metastatic breast cancer treated with pertuzumab, trastuzumab, and docetaxel: results from the randomized phase III study CLEOPATRA. Ann Oncol. 2014;25(6):1116-1121. doi:10.1093/annonc/mdu133 24685829 PMC4037862

[5] Lin NU, Borges V, Anders C, . Intracranial efficacy and survival with tucatinib plus trastuzumab and capecitabine for previously treated HER2-positive breast cancer with brain metastases in the HER2CLIMB trial. J Clin Oncol. 2020;38(23):2610-2619. doi:10.1200/JCO.20.00775 32468955 PMC7403000

[6] Bartsch R, Berghoff AS, Furtner J, . Trastuzumab deruxtecan in HER2-positive breast cancer with brain metastases: a single-arm, phase 2 trial. Nat Med. 2022;28(9):1840-1847. doi:10.1038/s41591-022-01935-8 35941372 PMC9499862

[7] Ramakrishna N, Anders CK, Lin NU, . Management of advanced human epidermal growth factor receptor 2-positive breast cancer and brain metastases: ASCO guideline update. J Clin Oncol. 2022;40(23):2636-2655. doi:10.1200/JCO.22.00520 35640075

[8] Patchell RA, Tibbs PA, Walsh JW, . A randomized trial of surgery in the treatment of single metastases to the brain. N Engl J Med. 1990;322(8):494-500. doi:10.1056/NEJM199002223220802 2405271

[9] Mintz AH, Kestle J, Rathbone MP, . A randomized trial to assess the efficacy of surgery in addition to radiotherapy in patients with a single cerebral metastasis. Cancer. 1996;78(7):1470-1476. doi:10.1002/(SICI)1097-0142(19961001)78:7<1470::AID-CNCR14>3.0.CO;2-X 8839553

[10] Lebow ES, Pike LRG, Seidman AD, Moss N, Beal K, Yu Y. Symptomatic necrosis with antibody-drug conjugates and concurrent stereotactic radiotherapy for brain metastases. JAMA Oncol. 2023;9(12):1729-1733. doi:10.1001/jamaoncol.2023.4492 37883079 PMC10603573

[11] Bander ED, El Ahmadieh TY, Chen J, . Outcomes following early postoperative adjuvant radiosurgery for brain metastases. JAMA Netw Open. 2023;6(10):e2340654. doi:10.1001/jamanetworkopen.2023.40654 37906192 PMC10618851

[12] Boire A, Burke K, Cox TR, . Why do patients with cancer die? Nat Rev Cancer. 2024;24(8):578-589. doi:10.1038/s41568-024-00708-4 38898221 PMC7616303

[13] World Medical Association. World Medical Association Declaration of Helsinki: ethical principles for medical research involving human subjects. JAMA. 2013;310(20):2191-2194. doi:10.1001/jama.2013.281053 24141714

[14] Wolff AC, Somerfield MR, Dowsett M, . Human epidermal growth factor receptor 2 testing in breast cancer: ASCO-College of American Pathologists guideline update. J Clin Oncol. 2023;41(22):3867-3872. doi:10.1200/JCO.22.02864 37284804

[15] Laakmann E, Witzel I, Neunhöffer T, . Characteristics of patients with brain metastases from human epidermal growth factor receptor 2-positive breast cancer: subanalysis of Brain Metastases in Breast Cancer Registry. ESMO Open. 2022;7(3):100495. doi:10.1016/j.esmoop.2022.100495 35653983 PMC9271494

[16] Noteware L, Broadwater G, Dalal N, . Brain metastasis as the first and only metastatic relapse site portends worse survival in patients with advanced HER2 + breast cancer. Breast Cancer Res Treat. 2023;197(2):425-434. doi:10.1007/s10549-022-06799-7 36403183

[17] Leone JP, Lee AV, Brufsky AM. Prognostic factors and survival of patients with brain metastasis from breast cancer who underwent craniotomy. Cancer Med. 2015;4(7):989-994. doi:10.1002/cam4.439 25756607 PMC4529337

[18] Bander ED, Yuan M, Reiner AS, . Durable 5-year local control for resected brain metastases with early adjuvant SRS: the effect of timing on intended-field control. Neurooncol Pract. 2021;8(3):278-289. doi:10.1093/nop/npab005 34055375 PMC8153823

[19] Niikura N, Hayashi N, Masuda N, . Treatment outcomes and prognostic factors for patients with brain metastases from breast cancer of each subtype: a multicenter retrospective analysis. Breast Cancer Res Treat. 2014;147(1):103-112. doi:10.1007/s10549-014-3090-8 25106661

[20] Schnurman Z, Mashiach E, Link KE, . Causes of death in patients with brain metastases. Neurosurgery. 2023;93(5):986-993. doi:10.1227/neu.0000000000002542 37255296

[21] Swain SM, Baselga J, Kim SB, ; CLEOPATRA Study Group. Pertuzumab, trastuzumab, and docetaxel in HER2-positive metastatic breast cancer. N Engl J Med. 2015;372(8):724-734. doi:10.1056/NEJMoa1413513 25693012 PMC5584549

[22] Ferraro E, Khan A, Plitas G, . Abstract PO3-04-07: characterization of response to first-line chemotherapy, trastuzumab, and pertuzumab among patients with de novo metastatic HER2-positive breast cancer. *Cancer Res*. 2024;84(9 suppl):PO3-04-07.

[23] Abouharb S, Ensor J, Loghin ME, . Leptomeningeal disease and breast cancer: the importance of tumor subtype. Breast Cancer Res Treat. 2014;146(3):477-486. doi:10.1007/s10549-014-3054-z 25038877

[24] Morikawa A, Jordan L, Rozner R, . Characteristics and outcomes of patients with breast cancer with leptomeningeal metastasis. Clin Breast Cancer. 2017;17(1):23-28. doi:10.1016/j.clbc.2016.07.002 27569275 PMC5266701

[25] Carausu M, Carton M, Darlix A, . Breast cancer patients treated with intrathecal therapy for leptomeningeal metastases in a large real-life database. ESMO Open. 2021;6(3):100150. doi:10.1016/j.esmoop.2021.100150 33984675 PMC8134714

[26] Murthy RK, Loi S, Okines A, . Tucatinib, trastuzumab, and capecitabine for HER2-positive metastatic breast cancer. N Engl J Med. 2020;382(7):597-609. doi:10.1056/NEJMoa1914609 31825569

[27] Montemurro F, Delaloge S, Barrios CH, . Trastuzumab emtansine (T-DM1) in patients with HER2-positive metastatic breast cancer and brain metastases: exploratory final analysis of cohort 1 from KAMILLA, a single-arm phase IIIb clinical trial. Ann Oncol. 2020;31(10):1350-1358. doi:10.1016/j.annonc.2020.06.020 32634611

[28] André F, Cortés J, Curigliano G, . A pooled analysis of trastuzumab deruxtecan in patients with human epidermal growth factor receptor 2 (HER2)-positive metastatic breast cancer with brain metastases. Ann Oncol. 2024;35(12):1169-1180. doi:10.1016/j.annonc.2024.08.2347 39241960

[29] Giantini-Larsen A, Abou-Mrad Z, Goldberg JL, . Postradiosurgery cystic degeneration in brain metastases causing delayed and potentially severe sequelae: systematic review and illustrative cases. J Neurosurg Case Lessons. 2023;5(6):CASE22462. doi:10.3171/CASE22462 36748750 PMC10550559

[30] Moss NS, Beal K, Tabar V. Brain metastasis—a distinct oncologic disease best served by an integrated multidisciplinary team approach. JAMA Oncol. 2022;8(9):1252-1254. doi:10.1001/jamaoncol.2022.1928 35862025 PMC9984120

[31] Moss NS, El Ahmadieh TY, Brown S, . Integrated multidisciplinary brain metastasis care reduces patient visits and shortens time to adjuvant irradiation. JCO Oncol Pract. 2022;18(11):e1732-e1738. doi:10.1200/OP.22.00258 36037413 PMC10166425

