# Supplementary material for: Survival Among Patients With ERBB2-Positive Metastatic Breast Cancer and Central Nervous System Disease
Source: JAMA Netw Open. 2025 Jan 31;8(1):e2457483. doi: 10.1001/jamanetworkopen.2024.57483 (PMC11786230; doi:10.1001/jamanetworkopen.2024.57483)
Supplement: Supplement 1. — eTable. Clinicopathologic Characteristics Associated With the Presence of ECM Prior to or Synchronous With CNS Disease at Metastatic Disease Diagnosis eFigure 1. Flow Diagram eFigure 2. Time to Development of CNS Disease From the Diagnosis of Primary Breast Cancer [file jamanetwopen-e2457483-s001.pdf]

Supplementary Online Content

Ferraro E, Reiner AS, Bou Nassif R, et al. Survival in patients with *ERBB2*-positive metastatic breast cancer and central nervous system metastasis. *JAMA Netw Open*. 2025;8(1):e2457483. doi:10.1001/jamanetworkopen.2024.57483

eTable. Clinicopathologic Characteristics Associated With the Presence of ECM Prior to or Synchronous With CNS Disease at Metastatic Disease Diagnosis
eFigure 1. Flow Diagram
eFigure 2. Time to Development of CNS Disease From the Diagnosis of Primary Breast Cancer

This supplementary material has been provided by the authors to give readers additional information about their work.

© 2025 Ferraro E et al. *JAMA Network Open*.

eTable: Clinico-pathological characteristics associated with the presence of ECM (prior or synchronous to CNS disease) at the time of diagnosis of metastatic disease.

**Characteristic**	**Active ECM at Brain Mets Dx, N = 199[1]**	**Only Brain Mets or ECM in CR at Brain Mets Dx, N = 73[1]**	**p-value[2]**
Sex			>0.99
Female	197 (99.0)	73 (100.0)	
Male	2 (1.0)	0	
Race/ethnicity			0.97
ASIAN	19 (9.6)	7 (9.6)	
BLACK	23 (11.6)	6 (8.2)	
OTHER	9 (4.5)	3 (4.1)	
UNKNOWN	15 (7.5)	5 (6.8)	
WHITE	133 (66.8)	52 (71.2)	
Stage at Diagnosis			**<0.001**
1	13 (6.5)	4 (5.5)	
2	35 (17.6)	23 (31.5)	
3	41 (20.6)	31 (42.5)	
4	110 (55.3)	15 (20.6)	
De Novo Met	110 (55.3)	15 (20.6)	**<0.001**
HER2 Status of the Primary Breast Cancer			0.68
Negative	1 (0.5)	0	
Positive	188 (94.5)	68 (93.2)	
Unknown	10 (5.0)	5 (6.9)	
ER and PR Status on metastasis (first site of ECM or BM)			0.28
Negative	89 (44.7)	38 (52.1)	
Positive	110 (55.3)	35 (47.9)	
HER2 Status on metastasis (first site of ECM or BM)			0.30
Negative	9 (4.5)	1 (1.4)	
Positive	190 (95.5)	72 (98.6)	
HER2 status on metastasis details			0.72
3+	153 (76.9)	59 (80.8)	
FISH amplified	26 (13.1)	7 (9.6)	
Unknown	20 (10.1)	7 (9.6)	
AntiHER2 in Early Stage			**<0.001**
Adjuvant	48 (24.1)	16 (21.9)	
Neoadjuvant	48 (24.1)	42 (57.5)	
No	103 (51.8)	15 (20.5)	
pCR			**<0.001**
No	37 (18.6)	24 (32.9)	
Yes	6 (3.0)	18 (24.7)	

© 2025 Ferraro E et al. *JAMA Network Open*.

Characteristic	Active ECM at Brain Mets Dx, N = 199[1]	Only Brain Mets or ECM in CR at Brain Mets Dx, N = 73[1]	p-value[2]
POD	5 (2.5)	0	
NA, did not receive neoadjuvant	151 (75.9)	31 (42.5)	
Prior Antibody Taxane			0.42
0	24 (12.1)	8 (11.0)	
1	175 (87.9)	64 (87.7)	
Unknown	0	1 (1.4)	
Lines before Brain Mets Dx			**<0.001**
Median (Range)	1.00 (0.00, 14.00)	0.00 (0.00, 5.00)	
Mean (SD)	2.02 (3)	0.3 (1)	
Unknown	1	1	
Switch Therapy	89 (65.9)	7 (63.6)	>0.99
Unknown	64	62	
KPS nearest Brain Mets Dx			0.058
60	0	3 (17.6)	
70	5 (13.5)	1 (5.9)	
80	8 (21.6)	5 (29.4)	
90	20 (54.1)	5 (29.4)	
100	4 (10.8)	3 (17.6)	
Unknown	162 (81.4)	56 (76.7)	
ECM Disease Status at time of Brain Mets Dx			**<0.001**
CR	0	9 (12.5)	
No Dx of ECD at time of Brain Mets Dx	1 (0.5)	56 (77.8)	
non-POD	67 (34.5)	0	
POD	126 (64.9)	7 (9.7)	
Unknown	5	1	
Symptoms at Brain Mets Dx			**0.002**
Altered MS	6 (3.0)	1 (1.4)	
Dizziness/vague	52 (26.1)	16 (21.9)	
Focal	33 (16.6)	13 (17.8)	
ICP/mass effect	34 (17.1)	21 (28.8)	
Mass effect + other symptoms	2 (1.0)	1 (1.4)	
No symptoms	52 (26.1)	6 (8.2)	
Seizure	14 (7.0)	14 (19.2)	
Unknown	6 (3.0)	1 (1.4)	
Number of Brain Mets			**0.001**
0	11 (5.5)	4 (5.5)	
1	36 (18.1)	34 (46.6)	
2	35 (17.6)	10 (13.7)	
3	15 (7.5)	3 (4.1)	
4	11 (5.5)	2 (2.7)	
5	9 (4.5)	2 (2.7)	

© 2025 Ferraro E et al. *JAMA Network Open*.

Characteristic	Active ECM at Brain Mets Dx, N = 199[1]	Only Brain Mets or ECM in CR at Brain Mets Dx, N = 73[1]	p-value[2]
mult	82 (41.2)	18 (24.7)	
Number of Brain Mets (dural)			0.80
0	170 (85.4)	66 (90.4)	
1	21 (10.6)	5 (6.9)	
2	4 (2.0)	1 (1.4)	
3	1 (0.5)	1 (1.4)	
6	1 (0.5)	0	
multiple	2 (1.0)	0	
Age at Primary Breast Cancer (years)			>0.99
Median (Range)	49 (22, 82)	51 (27, 74)	
Mean (SD)	50 (12)	50 (10)	
Age at Brain Mets (years)			0.58
Median (Range)	54 (29, 87)	53 (32, 76)	
Mean (SD)	53.5 (13)	52 (10)	

Notes:
1: n (%)
2: Fisher's exact test; Pearson's Chi-squared test; Wilcoxon rank sum test

© 2025 Ferraro E et al. *JAMA Network Open*.

eFigure 1: Flow diagram

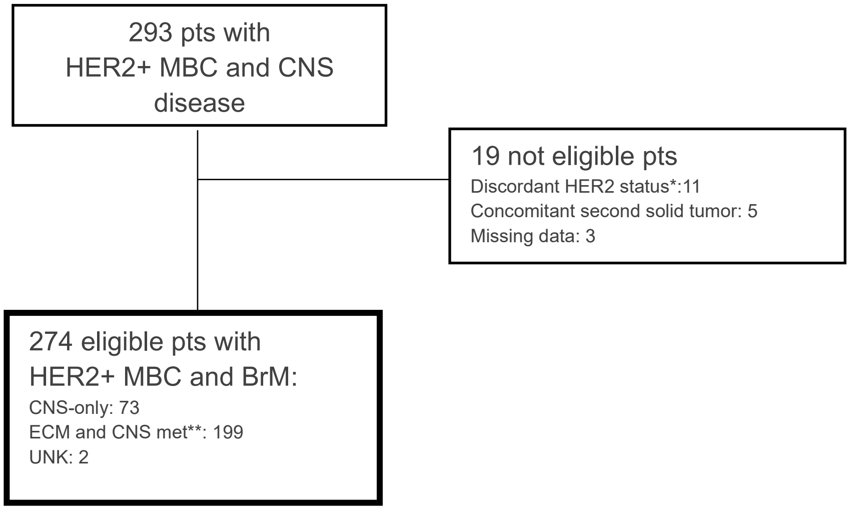

Notes:
*Discordant HER2 status is referring at discordance of HER2 status between primary and metastatic site or between metastatic sites. All these patients have never received anti-HER2 therapies in metastatic setting.
** Synchronous disease is defined as diagnosis of BrM and ECM in the time window +/-31 days

© 2025 Ferraro E et al. *JAMA Network Open*.

eFigure 2: Time to development of CNS disease from the diagnosis of primary breast cancer

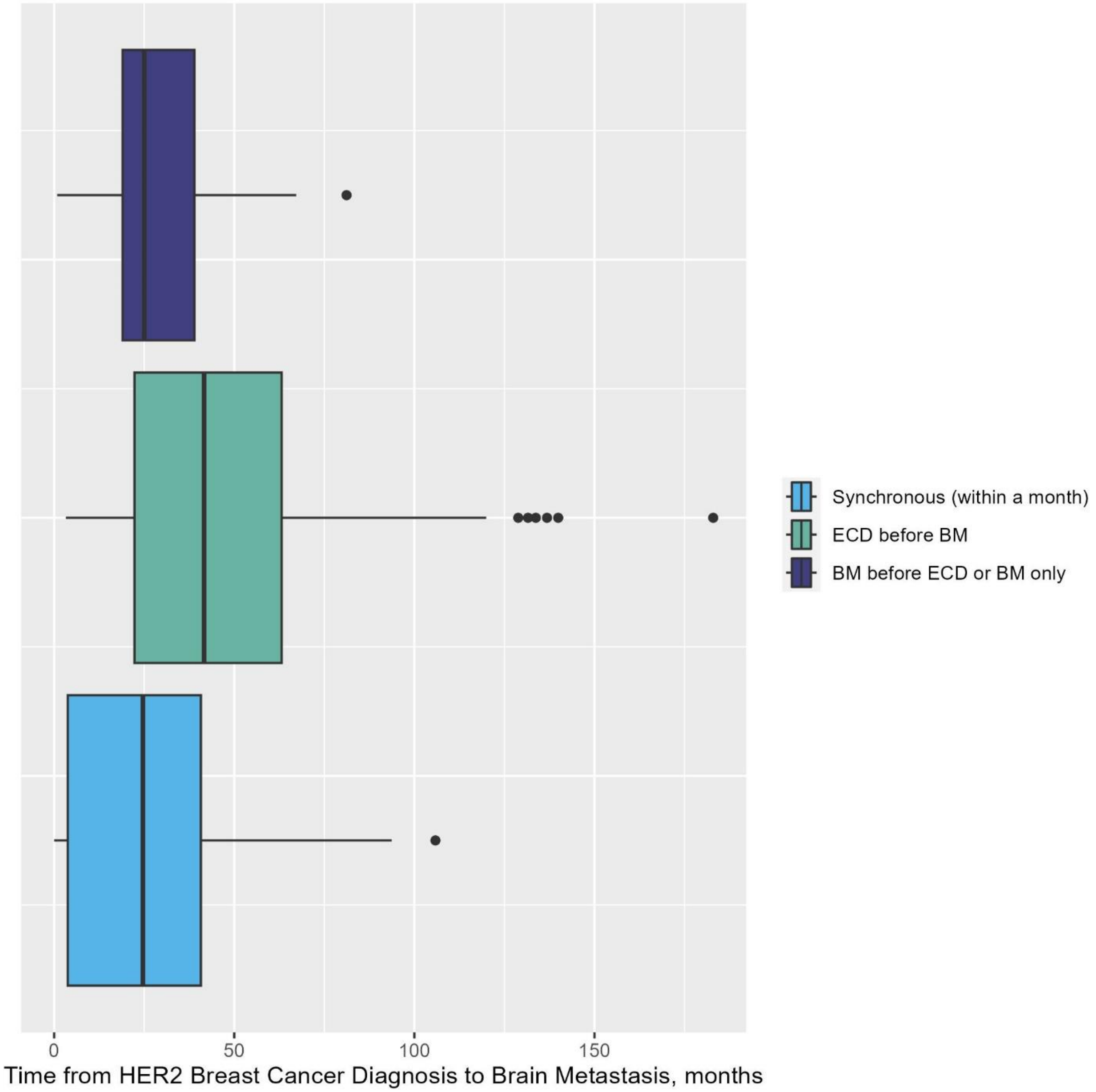

© 2025 Ferraro E et al. *JAMA Network Open*.
