# Supplementary material for: Survival Among Patients With ERBB2-Positive Metastatic Breast Cancer and Central Nervous System Disease
Source: JAMA Netw Open. 2025 Jan 31;8(1):e2457483. doi: 10.1001/jamanetworkopen.2024.57483 (PMC11786230; doi:10.1001/jamanetworkopen.2024.57483)
Supplement: Supplement 2. — Data Sharing Statement [file jamanetwopen-e2457483-s002.pdf]

Data Sharing Statement

Ferraro. Survival Among Patients With ERBB2-Positive Metastatic Breast Cancer and Central Nervous System Disease. *JAMA Netw Open*. Published January 31, 2025. doi:10.1001/jamanetworkopen.2024.57483

Data
Data available: No
